# Fidelity of an Online Telecoaching Community-Based Exercise Intervention Among Adults Living With HIV in Toronto, Ontario, Canada: Observational Longitudinal Study

**DOI:** 10.2196/84902

**Published:** 2026-09-03

**Authors:** Marfy Abousifein, Julia Nathanson, Tizneem Jiancaro, Ada Tang, Puja Ahluwalia, Ahmed M Bayoumi, Lisa Avery, George Da Silva, Francisco Ibáñez-Carrasco, Ivan Ilic, Kiera McDuff, Mona Loutfy, Annamaria Furlan, Zoran Pandovski, Helen Trent, Soo Chan Carusone, Kelly K O'Brien

**Affiliations:** 1 McMaster Collaborative for Health and Aging McMaster University Hamilton, ON Canada; 2 Department of Physical Therapy Temerty Faculty of Medicine University of Toronto Toronto, ON Canada; 3 School of Rehabilitation Science Faculty of Health Sciences McMaster University Hamilton, ON Canada; 4 Realize Toronto, ON Canada; 5 Institute of Health Policy, Management and Evaluation (IHPME) Dalla Lana School of Public Health University of Toronto Toronto, ON Canada; 6 MAP Centre for Urban Health Solutions St. Michael's Hospital Unity Health Toronto Toronto, ON Canada; 7 Department of Medicine Temerty Faculty of Medicine University of Toronto Toronto, ON Canada; 8 Biostatistics Department Princess Margaret Cancer Centre University Health Network Toronto, ON Canada; 9 Dalla Lana School of Public Health University of Toronto Toronto, ON Canada; 10 YMCA of Greater Toronto Toronto, ON Canada; 11 Women’s College Hospital Research Institute Women’s College Hospital Toronto, ON Canada; 12 Rehabilitation Sciences Institute (RSI) Temerty Faculty of Medicine University of Toronto Toronto, ON Canada

**Keywords:** exercise, fidelity, HIV/AIDS, implementation science, telerehabilitation

## Abstract

**Background:**

Community-based exercise (CBE) can prevent and mitigate disability and improve health outcomes in people aging with HIV; however, engagement in exercise can vary.

**Objective:**

This study aims to describe the fidelity of implementation (FOI) of an online telecoaching CBE intervention with adults living with HIV.

**Methods:**

We conducted an observational longitudinal study involving a 6-month online CBE intervention involving thrice-weekly 60-minute exercise sessions; online biweekly supervised one-on-one exercise sessions with a personal trainer (13 sessions); and online monthly group educational sessions. We assessed fidelity using (1) online structured interviews with participants living with HIV at months 2 and 6, comprising 20 fidelity items on trainer performance, participant engagement, and exercise experiences; and (2) coaching logs completed by personal trainers after biweekly training sessions (frequency, intensity, time, and type of physical activity during supervised sessions and self-reported physical activity in the prior week). We considered FOI criteria to be met if ≥80% of the participants living with HIV: (1) attended ≥80% of training sessions (11 of 13 sessions); (2) engaged in thrice-weekly exercise the week of the personal training session ≥80% of the time over the 6-month intervention, as reported on the coaching logs; (3) reported “complete criteria met” for ≥80% of the fidelity items (≥16 of 20 items) at month 2 and month 6, as reported from the interviews; and (4) engaged in a combination of aerobic, strength, balance, and flexibility exercise ≥80% of the time over the 6-month intervention, as reported in the coaching logs.

**Results:**

A total of 29 of 32 participants completed at least 1 fidelity interview; 69% (22/32) were male. FOI was met for 1 of our 4 criteria among the 18 participants who completed the intervention and FOI assessment at month 6, specifically criterion 1, whereby 83% (15/18; 95% CI 66%-100%) of participants attended ≥80% of the 13 coaching sessions. For criterion 2, 50% (9/18; 95% CI 27%-73%) of participants engaged in thrice-weekly exercise in the week of the personal training session ≥80% of the time over the 6-month intervention; criterion 3: ≥80% of participants reported criteria as “completely met” for 70% (14/20 items; 95% CI 50%-89%) of items at month 2 (n=27 participants) and 30% (6/20 items; 95% CI 10%-50%) of items at month 6 (n=18 participants); and criterion 4: 22% (4/18; 95% CI 3%-41%) of participants engaged in a combination of all 4 exercise types ≥80% of the time. Barriers to engagement included scheduling issues, lack of interest, technology problems, and episodic disability.

**Conclusions:**

FOI was achieved for supervised components of the CBE intervention and less so with independent exercise. Health and rehabilitation providers should tailor exercise interventions to personal preferences and abilities to improve engagement among adults living with HIV.

## Introduction

### Overview

In Canada, over 65,000 people were estimated to be living with HIV at the end of 2022 [1]. More individuals are living longer and aging with HIV; globally, persons living with HIV aged 50 years or older increased from 5.4 million in 2015 to 8.1 million in 2020 [2]. As people living with HIV age, they can experience health-related consequences of HIV, including an increased risk of cardiovascular disease, diabetes, bone and joint disorders, neurocognitive disorders, and certain cancers [3-5]. Rehabilitation, such as physical therapy and occupational therapy, can mitigate disability associated with HIV and multimorbidity, such as fatigue, pain, cognitive impairments, body composition changes, and employment challenges [6-9]. Exercise is a rehabilitation intervention and self-management strategy that can address disability and improve or sustain the health of individuals with HIV. Systematic review evidence indicates that exercise is safe and can lead to benefits in cardiopulmonary fitness, strength, weight and body composition, and psychological outcomes for people with HIV [10,11]. However, this evidence primarily involved supervised interventions by physical therapists and exercise physiologists that can be costly and unsustainable [10,11]. With up to an estimated 73% of people living with HIV classified as sedentary or physically inactive, it is essential to consider exercise interventions that are accessible and practical for people with HIV to sustain over the long term [12].

### Community-Based Exercise

Community-based exercise (CBE) is a promising approach that may help people living with HIV increase their engagement in exercise and physical activity [13-16]. Exercise is purposeful, planned, and repetitive bodily movement that has the objective of improving or maintaining physical fitness, while physical activity is any bodily movement that results in energy expenditure [17]. Persons living with HIV perceive benefits from exercise and physical activity as a component of health promotion, with the potential to improve physical function and mental health and reduce isolation [18]. CBE can be provided under the guidance of a health or fitness instructor, with the aim of encouraging consistent exercise and physical activity within the community [13,14,16]. CBE enhances the benefits of traditional exercise as it can improve social interaction, provide support and motivation, and promote emotional, cognitive, and behavioral self-management strategies to help individuals manage health challenges [14,19,20]. CBE has been associated with engagement in physical activity and improvements in health outcomes for adults living with HIV [19,21]; however, the factors influencing the implementation and uptake of CBE are less clear.

### Implementation of CBE

Using the Consolidated Framework for Implementation Research (CFIR), Cooper et al [22] conducted a qualitative systematic review of CBE interventions to explore barriers and facilitators of implementing CBE interventions in the general population [22]. Barriers included lack of participant motivation, insufficient resources allocated for implementation, the complexity of the CBE intervention, and lack of participant knowledge about the intervention [22]. Facilitators included the flexibility to adapt the intervention to the context, intervention compatibility with participants, and participants having a positive perception of those implementing the intervention [22]. The authors concluded that making minor changes to tailor an intervention while maintaining core components, such as clear implementation strategies and coordination of delivery, can facilitate the implementation of a CBE intervention.

Members of this authorship team assessed the impact of an in-person CBE intervention with adults living with HIV at the YMCA in Toronto, Ontario, Canada [21]. While the team reported improvements in cardiovascular health, strength, flexibility, and self-reported physical activity, barriers to taking part in CBE were documented among women, persons who are gender diverse, and persons who did not live geographically close to the YMCA [21]. Persons with HIV may experience obstacles to exercising in traditional gym environments, including interpersonal, financial, and geographical barriers, self-image issues and stigma, and difficulty initiating exercise following periods of inactivity [23,24].

### Telerehabilitation in the Context of HIV

Telerehabilitation, and more specifically telecoaching, is a potential strategy for implementing CBE with persons living with HIV. Telerehabilitation is the delivery of rehabilitation programs or services via technology, such as phone and video calls, web-based platforms, or mobile apps, used initially to increase efficiency, quality of care, and access to health care for populations with geographical [25,26], financial, or time-limiting barriers for persons living with HIV [27]. Telecoaching is a component of telerehabilitation that uses technology for remote supervision, guidance, and communication of an exercise program to improve physical activity levels and manage symptoms for people living with chronic diseases [28-33].

### Telecoaching CBE Study

We established an online telecoaching CBE intervention with the aim of enhancing engagement in physical activity among adults living with HIV in Toronto, Canada [34]. The intervention was developed based on foundational considerations for developing and implementing online CBE with adults living with HIV that included: (1) person-specific considerations (episodic nature of HIV, stigma, and HIV disclosure), (2) accessibility of the program, (3) program delivery and technology, (4) attributes of program personnel, (5) program content and design, and (6) building community and peer support [35].

Fidelity of implementation (FOI) is important to consider in online CBE interventions to develop practical interventions that lead to sustained physical activity. FOI, or integrity, is the degree to which programs are implemented as intended by the program developers [36-38]. The National Institutes of Health Behavior Change Consortium (BCC) treatment fidelity framework specifically refers to treatment fidelity and the link between training the providers, delivery, receipt, and enactment of the intervention for research involving health behavior interventions [39]. The fidelity with which an intervention is implemented can influence its level of success and uptake, and the impact on the outcome, with high fidelity promoting improved outcomes. However, it is important to consider planned adaptations to the intervention to optimize treatment fidelity while acknowledging the need to accommodate practical needs, study demands, and provider and participant burden, requiring pragmatic approaches in real-world settings [39]. Lack of fidelity can lead to failure in intervention implementation in a real-life context [40]. Hence, reporting fidelity is essential to determine the credibility, validity, and replicability of intervention study findings [41]. Despite the importance of understanding intervention fidelity, lack of knowledge or understanding about assessing fidelity may be barriers to assessment and reporting [42]. While quantitative and qualitative methods are recommended to comprehensively assess fidelity, this guidance is not consistently followed [39,42-44]. To our knowledge, there is limited evidence assessing the FOI of exercise interventions for persons living with HIV. Assessing the FOI of exercise interventions in the context of HIV is important because (1) sustained exercise can mitigate HIV-related disability, and (2) people living with HIV can face unique barriers and facilitators to exercise, such as episodic disability, which can influence the FOI of exercise interventions [45].

Our aim was to describe the FOI of a 6-month online telecoaching CBE intervention with adults living with HIV from the perspectives of participants (adults living with HIV) and personal trainers.

Findings from this study may help inform policy and programming for broader scale-up and optimal CBE implementation that can be adopted by fitness centers and community-based organizations to proactively prevent multimorbidity and improve health outcomes among persons living with HIV.

## Methods

### Study Design

**Figure 1:**
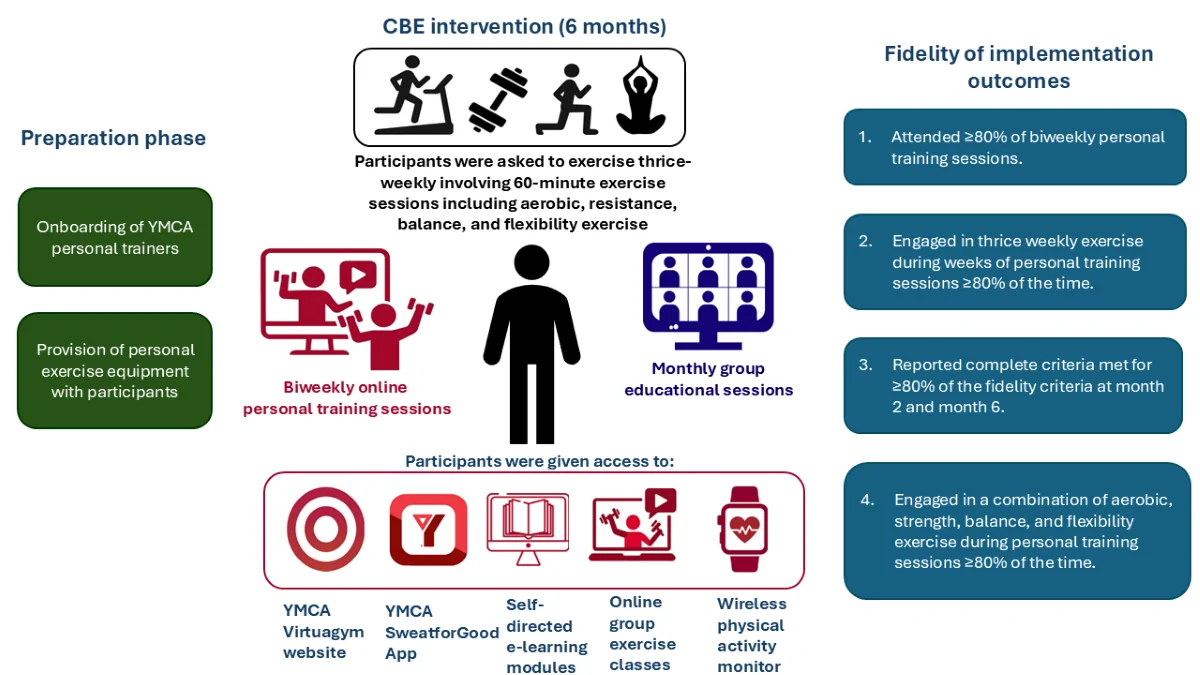
Overview of online community-based exercise (CBE) intervention and fidelity of implementation outcomes. YMCA: Young Men's Christian Association.

We conducted an observational longitudinal study involving (1) online structured interviews with adults living with HIV who participated in a 6-month online CBE intervention and (2) coaching logs of training sessions completed by personal trainers. Details of the study protocol have been previously published [34]. Figure 1 provides an overview of the CBE intervention and fidelity outcomes.

### Participants

We included adults living with HIV who participated in the telecoaching study and personal trainers from the YMCA who were involved in supervising personalized online exercise sessions with the study participants [34]. Eligibility criteria for the telecoaching study were adults (aged 18 years or older) who considered themselves safe to engage in exercise as determined by the self-administered Physical Activity Readiness Questionnaire (PAR-Q) and had access to an electronic device with an internet connection and a webcam, as well as space at home for exercise [34]. We recruited potential participants living with HIV through community-based organizations, an HIV clinic in Toronto via the Ontario HIV Treatment Network Cohort Study (OCS), and participants from a prior CBE study who agreed to be contacted about future research [21,46].

### Telecoaching Online CBE Intervention

Prior to the CBE implementation, fitness instructors (certified personal trainers and staff at the YMCA) took part in workshops on HIV, rehabilitation and exercise for adults living with HIV, goal setting, and research procedures [47]. We piloted the setup of the technology, including (but not limited to) Zoom software [48], a webcam, YMCA membership, web-based questionnaire software, and the Fitbit app [49] with the participants living with HIV and fitness instructors.

The telecoaching online CBE intervention included a 6-month structured intervention phase followed by an independent 6-month exercise phase [34]. During the 6-month intervention phase, participants were asked to engage in the following three components of the intervention: (1) an exercise regimen of aerobic, resistance, balance, and flexibility exercise occurring thrice weekly at moderate to vigorous intensity for ≥60 minutes per session; (2) attend online biweekly individually tailored one-on-one exercise sessions with a certified personal trainer from the YMCA, involving supervision and progression of aerobic, resistance, balance, and flexibility exercise; and (3) engage in online monthly group educational sessions on sleep, nutrition, goal setting, cognitive health, chronic pain, and other topics related to self-management, health, and physical activity while living with HIV, delivered by members of the research team. Each participant met with their personal trainer prior to the intervention to tailor the intervention to their goals, abilities, and interests [50]. Participants received a YMCA membership allowing them to access online YMCA classes through the YMCA SweatforGood app. Personal trainers uploaded the individualized exercise plans to the SweatforGood app, allowing participants to access, review, and track their exercises [34].

After the 6-month intervention, participants entered a 6-month follow-up independent exercise phase, during which they were asked to continue with thrice-weekly exercise. They continued to have access to the YMCA online membership, providing access to group online classes, and the SweatforGood app, but the study no longer provided one-on-one personal training sessions or monthly educational sessions.

Participants were given home exercise equipment (Therabands and a wooden step) and a wireless physical activity monitor (Fitbit Inspire 2) to monitor and encourage exercise during both the 6-month CBE intervention and the 6-month follow-up period. Participants documented their physical activity through a weekly online exercise log. Participants were given the Fitbit Inspire 2 and exercise equipment to keep at the end of the study as a token of appreciation for their participation. For more details, see the published protocol [34].

We aimed to recruit 40 adults living with HIV and 30 to complete the intervention, enabling us to achieve our primary study aim of assessing the impact of the CBE intervention [23,51].

### Data Collection

#### Overview

We used a combination of structured interviews with participants living with HIV and information collected in coaching logs completed by personal trainers to assess FOI.

#### Structured Fidelity Interviews: Perspectives of Persons Living With HIV

We conducted structured interviews with participants living with HIV to obtain perspectives on experiences with the CBE intervention and assess the extent to which the prescribed exercises were delivered according to the established program (FOI). Two members of the team conducted the interviews on Zoom. Interviews were approximately 10 minutes in duration and occurred partway through (month 2) and at the completion of the intervention (month 6). Participants were asked to share their experiences based on their most recent online personal training session and provided insights on any deviations from the prescribed regimen in an open-ended comment section. Responses were recorded electronically on an FOI form developed in Microsoft Word (Multimedia Appendix 1). Each question was scored by research staff as 0 (no criteria for fidelity were met), 1 (partial criteria met), or 2 (complete criteria met). For example, for the item regarding whether the “fitness provider provided direct and clear instructions during...[the] exercise session,” the item would be rated 0 if the participant stated that the instructions provided by the fitness provider were never clear or direct; 1 if the participant stated that the instructions provided by the fitness provider were sometimes clear and direct; or 2 if the participant stated that the fitness provider’s instructions were always clear and direct. TJ also documented open-ended responses from the participants to further describe barriers and facilitators to fidelity and provide additional context to the numeric ratings.

We evaluated FOI based on 20 items (defined a priori) captured in the interviews. The items were divided into four sections: (1) the completion of their exercise session within the allotted time (1 item), (2) their personal trainer’s performance during the session (7 items), (3) participant engagement and experience within the session (6 items), and (4) the broader online CBE study (6 items; Multimedia Appendix 1). FOI for a participant was defined as met if a participant met ≥80% (16/20) of the indicators at months 2 and 6 (from the perspective of the participant living with HIV). Overall, we considered FOI as met if ≥80% of the participants in the study met (achieved) ≥80% of FOI indicators (16/20 items; criterion 3).

#### Coaching Logs of Personal Training Sessions: Perspectives of Personal Trainers

After each supervised online training session with participants living with HIV, the personal trainers completed an online web-based questionnaire, referred to as the “coaching log.” Personal trainers were asked to document (1) whether the participants attended their biweekly personal training session; (2) whether they engaged in aerobic, strength, balance, and flexibility exercise during their biweekly personal training session; and (3) the number of days each participant exercised in the week prior to their session (Multimedia Appendix 2).

FOI for a participant was defined as met, from the perspectives of the personal trainers, if a participant attended ≥ 80% (11 out of 13) of their biweekly coaching sessions (criterion 1), if they exercised 3 times over the past week for ≥1 hour per session (criterion 2), and if they completed a combination of all 4 exercise components (aerobic, strength, balance, and flexibility) ≥80% of the time across their personal training sessions (criterion 4). Collectively, we considered FOI as met if ≥80% of the participants in the study achieved these 4 criteria (Table 1).

**Table 1:** FOI^a^ interpretation criteria.

FOI criteria for each participant^b^	Target population	Data source
Criterion 1: attended ≥80% of biweekly training sessions (at least 11 of 13) over the 6-month intervention	Personal trainers	Coaching logs
Criterion 2: engaged in thrice-weekly exercise the week of the personal training session ≥80% of the time over the 6-month intervention	Personal trainers	Coaching logs
Criterion 3: reported “complete criteria met” for ≥80% of the fidelity items (at least 16 of 20 items) at months 2 and 6 of the intervention	Participants living with HIV	Structured interview at months 2 and 6
Criterion 4: engaged in a combination of aerobic, strength, balance, and flexibility exercise during supervised personal training sessions ≥80% of the time over the 6-month intervention	Personal trainers	Coaching logs

^a^FOI: fidelity of implementation.

^b^Overall FOI was considered “met” for the community-based exercise intervention if the 4 criteria were achieved by ≥80% of participants.

#### Participant Demographic Questionnaire

We administered a web-based demographic questionnaire to participants living with HIV to collect baseline characteristics such as age, gender, time since HIV diagnosis, and race and ethnicity.

### Analysis

#### Overview

We entered numeric responses from the FOI form collected during the interviews, coaching logs, and demographic questionnaires into Microsoft Excel and analyzed the data using descriptive statistics [52]. We calculated frequencies and percentages for categorical variables and the median and IQR for continuous variables.

#### Structured Fidelity Interview

We reported the number and percentage of participants living with HIV who reported “no criteria met” (0), “partial criteria met” (1), and “complete criteria met” (2) for each of the 20 items assessed at months 2 and 6, based on the participants’ most recent training session (criterion 3). Open-ended responses from the structured interviews were analyzed using content analysis [53].

#### Coaching Logs

We calculated the median number of personal training sessions attended over the 6-month intervention (out of 13 sessions total). We calculated the number and proportion of participants who engaged in a combination of all 4 exercise types (aerobic, strength, balance, and flexibility) during the supervised coaching session. We calculated the number and proportion of participants who reported engaging in thrice-weekly exercise in the week of the personal training session, as recorded by the personal trainers in the coaching logs. We summed the number and proportion of participants who attended ≥80% (11/13) of biweekly coaching sessions (criterion 1), summed the number and proportion of participants who engaged in thrice-weekly exercise ≥80% of the time over the 6-month intervention (criterion 2), and the number and proportion of participants who completed the combination of all 4 exercise types in ≥80% of the sessions they attended (criterion 4) [54-56].

To account for attrition across the 6-month intervention, we reported baseline characteristics of participants at baseline and at months 2 and 6. We reported fidelity outcomes among participants who remained in the study at month 2 and who completed the intervention at month 6, as well as the original sample of participants who initiated the intervention.

### Fidelity Interpretation

We defined overall FOI of the CBE intervention as achieved if the following 4 criteria were met by ≥80% of the participants in the study: criterion 1, attended ≥80% (11/13) of their 13 training sessions; criterion 2, engaged in (at least) thrice-weekly exercise in the week of the personal training session ≥80% of the time over the 6-month intervention, as recorded by the personal trainers on the coaching logs; criterion 3, reported “complete criteria met” for ≥80% of the fidelity elements (at least 16 of 20 items) at months 2 and 6, as reported in the interviews with participants living with HIV; and criterion 4, engaged in a combination of aerobic, strength, balance, and flexibility exercises during the supervised coaching sessions ≥80% of the time over the 6-month intervention, as reported by the personal trainers in the coaching logs. Our criteria are based on the literature, whereby 80% to 100% is considered high fidelity, and ≤50% defines low FOI [54-56].

### Ethical Considerations

This study was approved by the University of Toronto Health Sciences Research Ethics Board (#40410). Participants provided informed verbal consent to participate in the study, which was documented on the consent form. Participant data were anonymized and stored on a secure server at the University of Toronto. Participants did not receive compensation for their participation in the exercise intervention. They were able to retain their exercise equipment and wireless physical activity monitor at study completion.

## Results

### Overview

Adults living with HIV who initiated the intervention and had at least 1 completed coaching log were included in the study. Of the 32 participants, 18 (56%) completed the 6-month intervention and had an FOI interview assessment. A total of 29/32 (91%) participants who completed at least 1 fidelity interview were included in the FOI analysis. Further, 16 (50%) participants completed the FOI assessment (interviews) at both months 2 and 6; 11 (34%) completed the assessment at month 2 only, and 2 (6%) completed the assessment at month 6 only. Of a potential 64 fidelity interviews, 45 (70%) were completed. At month 2, a total of 84% (27/32) of assessments were completed, and at month 6, a total of 56% (18/32) of assessments were completed.

Of the 32 participants at baseline, the majority were male and living with 2 or more concurrent health conditions in addition to HIV. Table 2 provides characteristics of participants enrolled in the study at baseline, month 2, and month 6.

**Table 2:** Characteristics of participants living with HIV at baseline and among those who completed an FOI^a^ interview at months 2 and 6.

Characteristic			Baseline (n=32)	Month 2 (n=27)		Month 6 (n=18)
Age (years), median (IQR)			53 (44-60)	50 (42-59)		57 (47-59)
**Sex, n (%)**						
	Male	22 (69)		18 (67)	13 (72)	
	Female	10 (31)		9 (33)	5 (28)	
**Gender, n (%)**						
	Man, “cis-man”	22 (69)		18 (66)	4 (22)	
	Woman, “cis-woman”	9 (28)		8 (30)	13 (72)	
	Nonbinary	1 (3)		1 (4)	1 (6)	
**Sexual orientation, n (%)**						
	Gay/homosexual	18 (56)		15 (56)	11 (61)	
	Heterosexual	7 (22)		7 (26)	3 (17)	
	Queer	4 (13)		3 (11)	2 (11)	
	Bisexual	1 (3)		1 (4)	1 (6)	
**Race/ethnicity^b^, n (%)**						
	White	14 (44)		11 (41)	6 (30)	
	South Asian	8 (25)		8 (30)	4 (20)	
	Black	8 (25)		6 (22)	5 (28)	
	Hispanic	2 (6)		2 (7)	2 (11)	
	Métis	2 (6)		1 (4)	0 (0)	
	Southeast Asian	1 (3)		1 (4)	1 (6)	
**Gross annual income (CAD $^c^), n (%)**						
	<20,000	7 (22)		0 (0)	5 (28)	
	20,000-49,999	17 (53)		20 (74)	7 (39)	
	>50,000	8 (25)		7 (26)	6 (33)	
Married or in a committed relationship, n (%)			14 (44)	13 (48)		7 (39)
Live alone, n (%)			12 (38)	7 (26)		7 (39)
Living with 2 or more concurrent health conditions in addition to HIV, n (%)			23 (72)	18 (67)		11 (61)

^a^FOI: fidelity of implementation.

^b^Participants could select more than 1 response.

^c^CAD $1=US $0.71 as of August 1, 2026.

Of the 32 participants, a total of 8 (25%) who initiated the intervention completed all 13 coaching sessions, 7 (24%) of whom were among the 29 participants who completed an FOI assessment interview. We received responses to 72% (298/416) of the coaching logs out of the total possible for 32 participants living with HIV to determine FOI from the perspectives of personal trainers.

### Fidelity Results

#### Overall Fidelity

FOI was met for attendance criterion 1 among participants who completed the intervention, whereby 83% (15/18; 95% CI 66%-100%) of participants attended ≥80% of their personal training sessions (Table 3). We describe results as they pertain to each criterion below.

**Table 3:** Summary of FOI^a^ criterion achievement.

FOI criterion	Data source	Participants who met the criteria among those who initiated the intervention (n=32), n (%); 95% CI	Participants who met the criteria among those who completed the intervention and month 6 FOI assessment (n=18), n (%) 95% CI	Fidelity met: achieved by ≥80% of participants (yes or no)
Criterion 1: attended ≥80% of biweekly personal training sessions (at least 11 of 13 sessions)	Coaching log attendance documented by personal trainers	18 (56); 39-73	15 (83); 66-100	Yes^b^
Criterion 2: engaged in thrice-weekly exercise the week of the personal training session ≥80% of the time over the 6-month intervention^c^	Coaching logs documented by personal trainers	11 (34); 18-50	9 (50); 27-73	No
Criterion 3: reported “complete criteria met” for ≥80% of the fidelity criteria (≥16 of the 20 items) at months 2 and 6^d^	Structured interview at months 2 and 6 with participants living with HIV	Month 2 (n=27): 21 (78) 62-93 and month 6 (n=18): 8 (44); 27-67	Month 2 (n=16): 12 (75); 54-96 and month 6 (n=18): 8 (44); 21-67	No
Criterion 4: engaged in a combination of aerobic, strength, balance, and flexibility exercises during supervised personal training sessions ≥80% of the time over the 6-month intervention	Coaching logs documented by personal trainers	8 (25) 10-40	4 (22) 3-41	No

^a^FOI: fidelity of implementation.

^b^Indicates that criterion 1 was met among participants who completed the intervention (≥80%).

^c^For criterion 2, the week of the first personal training session was excluded; baseline was defined as the start of the intervention.

^d^For criterion 3, the proportion of participants who selected “complete criteria met” for ≥80% of the fidelity criteria also were calculated based on the total number of participants who initiated the intervention (n=32). Participants who did not complete the fidelity assessments were assumed not to have met the criteria: At month 2, n=20/32 (63%; 95% CI 46%-79%); at month 6, n=8/32 (25%; 95% CI 10%-40%).

#### Criterion 1: Attended ≥80% of Personal Training Sessions by ≥80% of Participants

This FOI criterion was met for the participants who completed the intervention and an FOI assessment at month 6; 83% (15/18; 95% CI 66%-100%) of the participants attended ≥80% of the 13 coaching sessions (Table 3). Of all 32 participants, 18 (56%; 95% CI 39%-73%) attended ≥80% of the 13 coaching sessions (Table 3). Of all 32 (100%) participants who attended at least 1 personal training session, the median number of training sessions attended was 11 of 13 (IQR 6-12). Reasons for not attending or having to reschedule the online coaching sessions included scheduling issues, health reasons, or unknown reasons.

#### Criterion 2: Engaged in Thrice-Weekly Exercise the Week of the Personal Training Session ≥80% of the Time Over the 6-Month Intervention by ≥80% of Participants

This FOI criterion was not met. A total of 34% (11/32; 95% CI 18%-50%) of all participants and 50% (9/18; 95% CI 27%-73%) of those who completed the month 6 FOI assessment engaged in thrice-weekly exercise in the week of the personal training session ≥80% of the time over the 6-month intervention (Table 3). The median number of days of exercise per week for all participants (n=32) and for those who completed the intervention and month 6 FOI assessment (n=18) was 3 (IQR 3-5) and 4 days (IQR 3-5), respectively.

#### Criterion 3: Participants Living With HIV Reported ≥80% of Criteria as “Completely Met” by ≥80% of Participants

This FOI criterion, defined as ≥80% of participants reporting ≥80% of FOI elements (items) as “completely met,” was not achieved at months 2 or 6 (Table 3). Among the participants who completed an FOI assessment, 78% (21/27; 95% CI 62%-93%) and 44% (8/18; 95% CI 27%-67%) of the participants completely met the FOI elements for ≥80% of the 20 items at months 2 and 6, respectively. When considering the entire sample of 32 participants, fewer items met the criteria, whereby ≥80% of participants reported the FOI element as “completely met” for 4 of 20 items at month 2 and none of the 20 items at month 6 (details are provided in Multimedia Appendix 3).

Table 4 provides a detailed breakdown of the FOI assessment across the 20 interview items. At least 80% of participants reported the FOI element as “completely met” for 70% (14/20; 95% CI 50%-89%) of items at month 2 (n=27) and 30% (6/20; 95% CI 10%-50%) of items at month 6 (n=18). At month 2, the following 6 elements did not achieve fidelity at the threshold of ≥80% of participants: (1) participant completed their session in the allotted time (1 hour with coach online; 21/27, 78%; 95% CI 62%-94% of participants completely met this item), (2) participant responded to and completed the most recent weekly exercise email or SMS text message (20/27, 74%; 95% CI 58%-91%), (3) participant completed their additional individual independent exercise sessions (60 minutes twice per week, for a total of 60 minutes thrice per week) in the past week (13/27, 48%; 95% CI 29%-67%), (4) participant managed the required technology (eg, Zoom and SweatforGood app; 15/27, 55%; 95% CI 36%-74%), (5) participant engaged in monthly self-management sessions online (12/27, 45%; 95% CI 26%-63%), and (6) participant attended weekly group-based classes online at the YMCA (8/27, 30%; 95% CI 13%-47%; Table 4). At month 6, the following eight additional elements were not met: (1) fitness trainer instructed participant to complete resistive exercises according to their set exercise program (13/18, 72%; 95% CI 52%-93% of participants completely met this criterion), (2) fitness trainer instructed participant to complete flexibility exercises according to their set exercise program (13/18, 72%; 95% CI 52%-93%), (3) participant engaged in the prescribed strength exercises during the most recent session (12/18, 67%; 95% CI 45%-89%), (4) fitness trainer instructed participant to complete neuromotor/balance exercises according to their set exercise program (12/18, 67%; 95% CI 45%-89%), (5) participant engaged in the prescribed flexibility exercises (12/18, 67%; 95% CI 45%-89%), (6) fitness trainer instructed participant to complete aerobic exercises according to their set exercise program (11/18, 61%; 95% CI 39%-84%), (7) participant engaged in the prescribed neuromotor/balance exercises (11/18, 61%; 95% CI 39%-84%), and (8) participant engaged in the prescribed aerobic exercises during the most recent session (9/18, 50%; 95% CI 27%-73%; Table 4).

Content analysis of the open-ended responses identified barriers to meeting some of the criteria and coaching practices that were helpful for participants. The most common barriers to attending YMCA group-based classes were scheduling issues, lack of interest, and technology issues. Fitbit Inspire 2 wristband discomfort was cited as a barrier to wearing the physical activity monitor regularly. Illness, injury, pain, discomfort, and lack of interest were common barriers to completing the aerobic, resistance, balance, and flexibility exercises. Statements of appreciation for the personal training sessions included “[coach] demonstrates exercises to ensure participant understands proper form” (P16; month 2) and “[coach] demonstrates all exercises/modifications then participant performs, and trainer provides feedback” (P2; month 2). Overall, exercise demonstration, adaptation to participant circumstances, and providing feedback were positive coaching practices identified by participants.

**Table 4:** FOI^a^ (criterion 3)–structured interviews with adults living with HIV. “Fitness trainer” is analogous to “personal trainer.”

FOI item			Month 2 (n=27 interviews), %						Month 6 (n=18 interviews), %				
			No criteria met (0), n (%); 95% CI		Partial criteria met (1), n (%); 95% CI		Complete criteria met (2), n (%); 95% CI		No criteria met (0), n (%); 95% CI		Partial criteria met (1), n (%); 95% CI		Complete criteria met (2), n (%); 95% CI
**External factor^b^**													
	Item 1: participant completed their session within the allotted time (1 hour with a coach online)	0 (0)		6 (22); 7-38		21 (78); 62-94		0 (0)		4 (22); 3-41		14 (78); 27-61	
**Fitness trainer factors: the fitness trainer’s performance during the session^b^**													
	Item 2: fitness trainer provided direct and clear instructions during exercise session	0 (0)		0 (0)		27 (100)^c^		2 (11); 0-26		0 (0)		16 (89); 74-100^c^	
	Item 3: fitness trainer interacted with participant in a way that was meaningful or helpful for their session (provided eye contact, feedback, ongoing communication, etc)	0 (0)		2 (7); 0-17		25 (93); 83-100^c^		0 (0)		2 (11); 0-26		16 (89); 74-100^c^	
	Item 4: fitness trainer instructed participant to complete aerobic exercises according to their prescribed exercise program	2 (7); 0-17		3 (11); 0-23		22 (82); 53-84^c^		3 (17); 0-34		4 (22); 3-41		11 (61); 39-84	
	Item 5: fitness trainer instructed participant to complete resistive exercises according to their set exercise program	1 (4); 0-11		0 (0)		26 (96); 89-100^c^		2 (11); 0-26		3 (17); 0-34		13 (72); 52-93	
	Item 6: fitness trainer instructed participant to complete neuromotor/balance exercises according to their prescribed exercise program	1 (4); 0-11		1 (4); 0-11		25 (93); 83-100^c^		4 (22); 3-41		2 (11); 0-26		12 (67); 45-89	
	Item 7: fitness trainer instructed participant to complete flexibility exercises according to their prescribed exercise program	0 (0)		3 (11); 0-23		24 (89); 77-100^c^		3 (17); 0-34		2 (11); 0-26		13 (72); 52-93	
	Item 8: fitness trainer changed the program from previous sessions to progress the participant’s activity (either increase or decrease in intensity)	0 (0)		1 (4); 0-11		26 (96); 89-100^c^		1 (6); 0-16		0 (0)		17 (94); 84-100^c^	
**Participant factors: participant engagement and experience^c^**													
	Item 9: participant understood the instructions provided by their coach	0 (0)		2 (7); 0-17		25 (93); 83-100^c^		2 (11); 0-26		1 (6); 0-16		15 (83); 66-100^c^	
	Item 10: participant accepted direction/feedback provided by the coach	0 (0)		0 (0)		27 (100)^c^		2 (11); 0-26		0 (0)		16 (89); 74-100^c^	
	Item 11: participant engaged in the prescribed aerobic exercises during the most recent session	2 (7); 0-17		3 (11); 0-23		22 (82); 67-96^c^		3 (17); 0-34		6 (33); 12-55		9 (50); 27-73	
	Item 12: participant engaged in the prescribed resistive exercises during the most recent session	1 (4); 0-11		1 (4); 0-11		25 (93); 83-100^c^		2 (11); 0-26		4 (22); 3-41		12 (67); 45-89	
	Item 13: participant engaged in the prescribed neuromotor/balance exercises	1 (4); 0-11		1 (4); 0-11		25 (93); 83-100^c^		4 (22); 3-41		3 (17); 0-34		11 (61); 39-84	
	Item 14: participant engaged in the prescribed flexibility exercises	1 (4); 0-11		1 (4); 0-11		25 (93); 83-100^c^		3 (17); 0-34		3 (17); 0-34		12 (67); 45-89	
**Additional factors: the broader telecoaching study intervention**													
	Item 15: participant completed additional individual sessions (60 minutes twice per week) during the past week	5 (19); 4-33		9 (33); 15-51		13 (48); 29-67		4 (22); 3-41		3 (17); 0-34		11 (61); 39-84	
	Item 16: participant responded to or completed the most recent weekly exercise email/text message	6 (22); 7-38		1 (4); 0-11		20 (74); 58-91		4 (22); 3-41		2 (11); 0-26		12 (67); 45-89	
	Item 17: participant attended weekly group-based classes online at the YMCA	15 (56); 37-74		4 (15); 1-28		8 (30); 13-47		8 (44); 21-67		4 (22); 3-41		6 (33); 12-55	
	Item 18: participant engaged in monthly self-management sessions online	5 (19); 4-33		10 (37); 19-55		12 (45); 26-63		7 (39); 16-61		7 (39); 16-61		4 (22); 3-41	
	Item 19: participant wore a Fitbit regularly	0 (0)		3 (11); 0-23		24 (89); 77-100^c^		0 (0)		0 (0)		18 (100)^c^	
	Item 20: participant managed the required technology (Zoom, SweatforGood app, etc)	0 (0)		12 (44); 26-63		15 (55); 36-74		1 (5); 0-16		7 (39); 16-61		10 (56); 33-79	

^a^FOI: fidelity of implementation.

^b^Participants were asked about their experiences at their most recent personal training session.

^c^Indicates ≥80% of participants ‘completely met’ the criteria.

## Discussion

### Principal Findings

Among participants who completed the intervention and FOI assessment at month 6 (n=18), FOI was achieved for 1 of the 4 criteria, whereby 83% (95% CI 66%-100%) of participants who completed the study engaged in ≥80% of biweekly online personal training sessions. However, fidelity criteria were not met for independent exercise thrice weekly (50%; 95% CI 27%-73%), participant-reported fidelity criteria at month 6 (“complete criteria met” for at least 16/20, 44% of items; 95% CI 21%-67%), or engagement in all 4 exercise types in ≥80% of sessions (4/18, 22%; 95% CI 3%-41%).

Within the training session, the majority of participants engaged in strength and flexibility exercises, followed by aerobic and then balance exercise. This may be attributed to strength training requiring a higher level of supervision as opposed to aerobic activity that was done independently outside the formal personal training session. In the absence of equipment for aerobic activity, such as a treadmill or stationary bike, participants may have engaged in this type of activity independently outdoors.

Fidelity was achieved for some components of the intervention. At least 80% of participants completely met 14 out of 20 FOI elements at month 2 and 6 out of 20 FOI elements at month 6, as reported in the structured interviews. The FOI items least commonly met were independent engagement in exercise (for a total of thrice weekly or more of 60 minutes or longer), attendance at weekly group-based online classes at the YMCA (8/27, 29% of participants met this criterion), and attendance at monthly online group-based educational sessions (12/27, 45% of participants met this criterion).

### Supervised vs Independent Forms of Exercise Intervention

Low engagement in the online group-based YMCA classes and independent exercise outside of supervised one-on-one personal training sessions may be influenced by the unsupervised components of the intervention, as participants had higher engagement rates when supervised (ie, personal training sessions). This is consistent with the literature, which shows increased engagement in supervised exercise programs, with adults aged 65 years or older favoring supervised exercise programs [57]. As found within the open-ended FOI responses in this study, receiving feedback, education, and modified exercise tailored to participant preferences and abilities may be important factors driving adherence to supervised exercise [57-60]. The identification of the least-implemented intervention components can help inform future intervention design and may also inform the supports necessary for participants to sustain regular exercise routines postintervention. This may help to identify components of an intervention that require supervision and suggests the importance for future interventions to tailor CBE based on personalized preferences to promote engagement. Further, there is a need to explore population-specific factors that impact adherence to unsupervised exercise to better develop strategies that improve long-term behavioral change for adults living with HIV.

### Group-Based vs Individual Exercise Intervention

FOI criteria were not met for the group-based elements of the intervention. At month 2, a total of 45% (12/27; 95% CI 26%-63%) of participants reported attending the monthly online self-management sessions (≥80% of the time), and 22% (4/18; 95% CI 3%-41%) at month 6 (item 18 in the FOI assessment). At month 2, a total of 29% (8/27; 95% CI 12%-47%) of participants reported attending weekly online group-based exercise classes at the YMCA, and 33% (6/18; 95% CI 12%-55%) at month 6 (item 17 in the FOI assessment; Table 4). With HIV stigma being a barrier to seeking treatment, care, and health- and wellness-related programs, the lower engagement in group-based interventions may have been attributed to fear of HIV disclosure and discrimination [61-64].

### The Influence of Environmental and Personal Factors

Personal and environmental factors may further explain the FOI findings in this study. HIV is characterized by episodic disability, where there are unpredictable periods of wellness and illness [65]. Barriers to engaging in the intervention, as reported by participants, included illness, pain, or injury. The fluctuating nature of chronic conditions like HIV may make it challenging for participants to follow highly rigid schedules and training plans if their health and ability to engage in exercise vary day-to-day [23,51]. Perhaps our FOI criteria were too rigid and did not align with or consider the episodic and fluctuating nature of HIV. We recommend providers adapt the frequency and type of exercise to the nature of the illness to maintain the well-being of an individual living with HIV. Our findings indicate the need for flexibility with CBE interventions, enabling persons to cancel or reschedule sessions due to illness more frequently and engage in exercise at home [23,51]. We recommend that exercise interventions in this population be adaptable and considerate of the dynamic nature of the illness experience to ensure that intervention studies are pragmatic and translational to the real world [23,51,60].

While participants were provided with a YMCA online membership at no cost and access to a personal trainer biweekly, their financial situation still may have influenced engagement in the intervention. Income is positively correlated with exercise adherence [66,67]. Qualitative insights from this telecoaching CBE study reported that participants in this study highlighted cost as a limiting factor to engagement in physical activity [60]. A lower socioeconomic status may be correlated with poor exercise adherence due to a lack of leisure time. Individuals of higher socioeconomic status, on average, allocate more leisure time to exercise than those of lower socioeconomic status [66,67]. An association exists between low socioeconomic status and exercise in persons living with HIV, with barriers to exercise including caregiver responsibilities, time dedicated to childcare, unsafe neighborhoods, strict work schedules, and living in temporary dwellings [68,69]. Traditional exercise interventions may not be suitable for those living with HIV who are socioeconomically disadvantaged because of barriers related to employment, financial constraints, stigmatization, and inaccessible or lack of transportation, requiring interventions to be tailored to the needs of this population [68,69]. Collectively, it is important for health and fitness trainers to consider the environmental and personal factors that can influence exercise engagement and intervention adherence to tailor interventions accordingly. This may include tailoring modes of exercise delivery (eg, online and in-person; individual training or group-based classes) based on individual preferences.

### Strengths: Assessing Fidelity From 2 Different Perspectives

Strengths of our approach included our multimethod data collection at multiple time points of the CBE intervention and engaging perspectives of both participants living with HIV and personal trainers involved in implementation of the intervention. FOI in our study was assessed more positively from the perspectives of the participants living with HIV (through structured interviews), as opposed to the personal trainers’ logs of the coached sessions. Our fidelity assessment from the perspectives of participants living with HIV pertained to their most recent personal training session, whereas fidelity assessment from the perspective of the personal trainers captured engagement in all coached training sessions and the independent exercise done in the preceding week. Using multiple methods to assess intervention fidelity provided a greater understanding of fidelity [70,71] and allowed for different populations to have different perspectives on a shared phenomenon [37,44,70]. Toomey et al [71] recognized that disagreements between results of different fidelity assessment approaches from providers and persons with lived experiences may occur. Some of the differences in FOI findings in our study may be due to participants’ overestimation of their performance when completing self-reported measures (2 time points), as compared to the personal training coaching logs completed by providers more frequently across the biweekly supervised sessions (13 time points). Nevertheless, our assessment captured perspectives from both persons living with HIV and the trainers at different stages of the intervention.

### Limitations

Our study protocol included methods for capturing the implementation of the intervention components but did not explicitly state the definition of implementation fidelity. Prior to the analysis, we defined the FOI threshold most commonly reported in the literature as achievement of ≥80% of criteria [54-56]. We considered all 4 criteria for FOI to be weighted equally; however, in reality, specific aspects of the CBE intervention may be more important (eg, attendance at biweekly training sessions) to achieve FOI than others (ie, engaging in all 4 types of exercise in the training session). Given that we did not collect fidelity data in the same manner for both target populations (persons living with HIV and personal trainers), we were unable to directly compare the influence of perspectives on fidelity assessment. Furthermore, participants were asked about fidelity based on their most recent coaching session, meaning that a number of sessions were not captured in this analysis, nor do we have the reasons for noncompletion of the FOI interviews or coaching logs. We did not report physical activity as measured by the wireless physical activity monitor, as this was beyond the scope of this study. However, as the Fitbit was a component of the intervention, our FOI assessment included Fitbit usage (item 19 on the FOI assessment), whereby 89% to 100% of participants at months 2 and 6 wore the wireless physical activity monitor regularly. The uptake and usage of the Fitbit aligns with experiences of technology uptake and usage in the telecoaching CBE study [72].

Our study is limited to participants who remained in the study and completed the FOI assessment interview at month 6. Of 32 participants, only 18 (56%) completed the intervention and an FOI assessment at month 6, resulting in an overestimation of FOI. In our sensitivity analyses, assuming participants who withdrew or were lost to follow-up did not meet the criteria, only 18 (56%; 95% CI 39%-73%) achieved the criteria of engaging in ≥80% of biweekly online personal training sessions (Table 3 and Multimedia Appendix 3), resulting in none of the 4 FOI criteria being met for the study.

Another limitation was that the majority of participants living with HIV in this study identified as males and men; hence, findings may not be transferable to individuals who identify as females or women living with HIV. Also, we did not assess the association of contextual factors such as level of income, stigma, and discrimination. Future research should consider the intersecting components of sex, gender, culture, socioeconomic status, and stigma and their influence on engagement in exercise [73].

### Recommendations

There is a need for balancing FOI with personalized and tailored implementation of interventions for adults living with HIV [74-76]. While fidelity is important, solely prioritizing the implementation of the intervention as intended at the cost of tailoring the intervention to the population may not be the most beneficial for the population of adults living with HIV. To ensure the integrity and quality of intervention fidelity assessments while adapting the intervention to the population and its needs, it is important to define, a priori, the intervention components that may be modified to meet participant needs and circumstances. These include optimal frequency of supervised sessions (weekly vs biweekly), mode of exercise, and exercise setting to support independent exercise.

### Conclusions

FOI was achieved for the supervised personal training components of the CBE intervention but was not achieved for independent exercise among adults living with HIV who engaged in this 6-month online CBE intervention. Future research should consider tailoring online CBE interventions to personal preferences to foster uptake and sustained independent engagement in exercise over the long term.

## Appendix

Multimedia Appendix 1 Fidelity of implementation (FOI) assessment.

Multimedia Appendix 2 Personal training coaching log for personal trainers.

Multimedia Appendix 3 Fidelity of implementation (FOI) interviews for participants who initiated the study and completed month 2 and month 6 FOI interview assessments.

## Abbreviations

BCC: National Institutes of Health Behavior Change Consortium
CBE: community-based exercise
CFIR: Consolidated Framework for Implementation Research
FOI: fidelity of implementation
OCS: Ontario HIV Treatment Network Cohort Study
PAR-Q: Physical Activity Readiness Questionnaire

## Data Availability

Results of the study are supported by the data provided in the manuscript and its supplementary files. The complete dataset is available upon reasonable request from the corresponding author.
