# Supplementary material for: Fidelity of an Online Telecoaching Community-Based Exercise Intervention Among Adults Living With HIV in Toronto, Ontario, Canada: Observational Longitudinal Study
Source: JMIR Rehabil Assist Technol. 2026 Sep 3;13:e84902. doi: 10.2196/84902 (PMC13586703; doi:10.2196/84902)
Supplement: Supplementary file 1 [file rehab_v13i1e84902_app1.pdf]

Fidelity of Implementation (FOI) Assessment
(Completed by Research Coordinator)

Supplemental File 1– Fidelity of Implementation (FOI) of Tele-Coaching CBE Intervention

Month 2 and Month 6

Telephone / Zoom Script:

Hi [Participant Name],

I am [connecting with you] from the University of Toronto as part of the CBE Study to informally check-in with you about your experience with the Tele-Coaching CBE Study thus far. This is a casual conversation to specifically learn more about your **most current experience at with your YMCA Fitness Coaching Session** and the extent to which the exercise that **was actually prescribed to you is being delivered according to your established program** (i.e., ensuring that elements of implementation fidelity are addressed, consistently). We are asking you to share your experiences at each stage of the intervention midway (~Month 2), and upon competition of intervention (Month 6)). This interview (in-person or phone call) should take no more than 10 minutes.

We are trying to determine the extent to which:

1) Your fitness coach is administering the exercise plan as intended by our study, and
2) You are actually doing the exercises as prescribed

Your overall input will be used for the **future refinement** of an accessible, feasible, safe, and sustainable online CBE program for people living with HIV.

This phone call will not be audio-recorded. As member of the research team, I will be going through a brief checklist in order to document your feedback. I will not use any information that could identify you in any of the summaries that come from this conversation. There is no right or wrong answers we ask that you try to answer to the best of your ability.

A) Do you have 10 minutes so I can learn more about your overall experiences with the study?

- ❑ Yes
- ❑ No, not at this time but you can call back: [*insert date and time*] ____________________
- ❑ No – I do not wish to take part in this interview.

Before we start, do you have any questions?

Participant ID: _________

Fidelity of Implementation (FOI) Assessment
(Completed by Research Coordinator)

Intervention timeframe:

❑ Midway - Months 2-4 into intervention (Month: ____________)

❑ End of CBE Intervention Month 6

Date: _________________ Coaching Session # (1-13)_________ /25 week Research Staff Initials: _______

Start:

"In order to understand your most recent experience with your YMCA tele-coach, could you describe what exactly happened. For example, you had an exercise session with your fitness coach on **[dd/mm/yy]**. Can you walk me through from the time you connected online with your YMCA tele-coach to when you disconnected with your tele-coach?" [*Enter point form of the response in the notes/comments in section below*]

The following checklist items / questions refer to the participant's <u>most recent coaching session</u> on **[dd/mm/yy]** with [*INSTRUCTOR*]:

*Note – *if the participant does not describe / address each of the below criteria with the open-ended question above, the research staff may probe to explore the extent to which specific criteria were met*.

**EXTERNAL FACTORS**<br>Based on the participant open-ended description of the exercise session experience, to our knowledge…	**No Criteria Met**	**Partial Criteria Met**	**Complete Criteria Met**
1) The participant completed their session **in the allotted time** (1 hour with coach online)	❑	❑	❑
Comments:			
**FITNESS PROVIDER FACTORS**<br>Based on the participant open-ended description of the exercise session experience, to our knowledge, the fitness providers appeared to…	**No Criteria Met**	**Partial Criteria Met**	**Complete Criteria Met**
2) Fitness provider provided **direct and clear instructions** during your exercise session	❑	❑	❑

Fidelity of Implementation (FOI) Assessment
(Completed by Research Coordinator)

3) Fitness provider **interacted** with participant in a way that was **meaningful or helpful** for their session (e.g. provided eye-to-eye contact, feedback, ongoing communication, etc.)	❑	❑	❑
4) Fitness Provider **instructed** participant to complete ***aerobic*** exercises according to their set exercise program?	❑	❑	❑
5) Fitness Provider **instructed** participant to complete ***resistive*** exercises according to their set exercise program	❑	❑	❑
6) Fitness Provider **instructed** participant to complete ***neuromotor/balance*** exercises according to their set exercise program	❑	❑	❑
7) Fitness Provider **instructed** participant to complete ***flexibility*** exercises according to their set exercise program.	❑	❑	❑
8) Fitness Provider **changed the program** from past sessions to progress participant activity (either increase or decrease in intensity)	❑	❑	❑
Comments:			

**PARTICIPANT FACTORS**<br>Based on the participant open-ended description of the exercise session experience, to our knowledge, the participant appeared to show…	**No Criteria Met**	**Partial Criteria Met**	**Complete Criteria Met**
9) **Understanding of the instructions** provided by their coach	❑	❑	❑
10) **Accept direction/feedback** provided by the coach	❑	❑	❑
11) **Engage** in the prescribed **aerobic exercises** during the most recent session	❑	❑	❑
12) **Engage** in the prescribed **resistive exercises** during the most recent session	❑	❑	❑
13) **Engage** in the prescribed **neuromotor/balance exercises** during the most recent session	❑	❑	❑
14) **Engage** in the prescribed **flexibility exercises** during the most recent session	❑	❑	❑
Comments:			

Fidelity of Implementation (FOI) Assessment
(Completed by Research Coordinator)

**ADDITIONAL FACTORS**<br>The following questions / items refer to the broader CBE intervention. If not discussed in the open-ended question above, the research staff will ask participants the following about the broader CBE intervention…	**No Criteria Met**	**Partial Criteria Met**	**Complete Criteria Met**
15) **Complete** your **additional individual sessions** (min 2x/week) this past week?	❑	❑	❑
16) **Respond/complete** the most recent **weekly exercise email/text message?**	❑	❑	❑
**17) Attend weekly group based classes online at the YMCA**	❑	❑	❑
18) **Engage** in **monthly self-management sessions online**?	❑	❑	❑
19) **Wear your Fitbit regularly**?	❑	❑	❑
**20) Ability to manage the technology (Zoom, SweatforGood App, etc) for the intervention online**	❑	❑	❑
Comments:			
