# Supplementary material for: Fidelity of an Online Telecoaching Community-Based Exercise Intervention Among Adults Living With HIV in Toronto, Ontario, Canada: Observational Longitudinal Study
Source: JMIR Rehabil Assist Technol. 2026 Sep 3;13:e84902. doi: 10.2196/84902 (PMC13586703; doi:10.2196/84902)
Supplement: Supplementary file 2 [file rehab_v13i1e84902_app2.pdf]

Supplemental File 2: Personal Training Coaching Log for Personal Trainers

Initial YMCA Personal Fitness Training Session (Session #1/13)

Biweekly Fitness Personal Training Coaching Log – to be completed by the YMCA Staff Personal Trainers on Qualtrics at Each Training Session (Biweekly)

Please complete for your participants regardless of whether or not they attended their training session.

**Fitness Session Coaching Log for each session (Sessions #1 – 13)**<br>***There will be 13 Online Virtual Fitness Session Coaching Logs in Total**	
**Participant First Name and Last Initial**	
**Date of Initial YMCA Intake Fitness Session (#1):**	
**Main Health and Fitness Goal (SMART goal) at Intake**	
**Session # (2-13)**	
1. What is the CBE Tele-Coaching Study Participant ID #?	Enter the number only (without P).
2. What training session is this coaching log completed for?	Choose 1 to 13
3a) Did the participant attend a coaching session this week?	Yes<br>No<br>If no, reasons did not attend
Conditional Item – If No to 3a<br>3b) Did the participant provide at least 24 hours notice for cancelling their session?	Yes and the session was rescheduled<br>Yes, however the session was not rescheduled – Provide reason<br>No, therefore the session was not rescheduled.
4. What was the date of this week's coaching session?	Enter a date
5. What technology did the participant use to participate in the biweekly training session?	Laptop with webcam<br>Tablet<br>Other – please specify if known
6a) Were there any challenges or problems with the technology during the training session?	Yes<br>No<br>If Yes - 6b) Please describe the **technology challenges** during the session.
7. What was the participant's main health and fitness goal at intake?	Text entry
8a) Were there any changes in the participant's main health and fitness goal from intake at this session?	Yes – Please Describe: __________<br>No
8b) Were there any changes in health status of the participant since the last personal training session?	Yes – Please Describe: __________<br>No

Supplemental File 2: Personal Training Coaching Log for Personal Trainers

	**Aerobic / Cardio Exercise**
9a) Did the participant engage in at least one aerobic/cardio exercise during this session?	Yes<br>No – Please provide reason if applicable __________
9b) Please add any additional notes / details re: the aerobic activity completed during this session:	Text entry
	**Strength Exercise**
10a) Did the participant engage in at least one strength exercise during this session?	Yes<br>No – Please provide reason if applicable __________
10b) Please add any additional notes / details re: the strength exercise(s) completed during this session:	Text entry
	**Balance Exercise**
11a) Did the participant engage in at least one balance exercise during this session?	Yes<br>No – Please provide reason if applicable __________
11b) Please add any additional notes / details re: the balance exercise(s) performed during this session:	Text entry
	**Flexibility / Stretching Exercise**
12a) Did the participant engage in at least one flexibility/stretching exercise during this session?	Yes<br>No – Please provide reason if applicable __________
12b) Please add any additional notes / details re: the flexibility/stretching exercise(s) performed during this session:	Text entry

The following questions will be asked at each of the biweekly personal training sessions to the coaches to document the nature, extent and progression of the CBE program.

13. **<u>How many days</u>** did the participant exercise over the past week (out of the last 7 days)? *Choose one option.*

- ☐ 0 (None)
- ☐ 1 day
- ☐ 2 days
- ☐ 3 days
- ☐ 4 days
- ☐ 5 days
- ☐ 6 days
- ☐ 7 days

Supplemental File 2: Personal Training Coaching Log for Personal Trainers

14. Did the participant exercise **3 times over the past week** for **at least 1 hour per session**?

- ☐ Yes
- ☐ No (conditional, go to 14b)

Conditional question, if above is 'No'

14b. If the participant did not exercise 3 times over the past week for 1 hour per session what were the **reasons**?

____

15. Did the participant exercise as **part of a group** (with 2 or more other people) in the past week (past 7 days)? – this includes **Virtual YMCA Group Classes** (e.g. an exercise class or group activity)

- ☐ Yes
- ☐ No

Questions 16 and 17 relate to **The Canadian 24-Hour Movement Guidelines for Adults.** The guidelines state that to achieve health benefits, adults (aged 18-64 years) should engage in **moderate to vigorous intensity aerobic physical activities such that there is an accumulation of at least 150 minutes per week**.

The guidelines also state that adults should engage in **muscle strengthening activities using major muscle groups at least 2 days per week and several hours of light physical activities, including standing.**

Additionally, older adults (64+ years old) should engage in physical activities that **challenge balance.**

To learn more, please visit the link: https://csepguidelines.ca/

Moderate-intensity physical activities cause adults to sweat a little and to breathe harder (e.g., brisk walking, bike riding) and can be considered **4 to 5 out of 10** on the rate of perceived exertion (RPE) scale.

Vigorous-intensity physical activities cause adults to sweat and be 'out of breath' (e.g., jogging, **carrying or lifting,** cross-country skiing) and can be considered a **6 or more out of 10** on the rate of perceived exertion (RPE) scale.

Supplemental File 2: Personal Training Coaching Log for Personal Trainers

**0-10 Borg Rating of Perceived Exertion Scale Used to Measure the Intensity of Your Exercise**	
**0**	**Rest** / Nothing At All (e.g. lying on the couch)
**1**	**Really Easy** / Light (e.g. easy household chores)
**2**	**Easy** (e.g. leisurely walking that does not increase your heart rate)
**3**	
**4**	**Moderate** / Somewhat Hard (e.g. brisk walking or a moderate activity that speeds up your heart rate without making you significantly out of breath)
**5**	
**6**	**Hard** (e.g. vigorous activity, such as jogging or swimming, that increases your heart rate and makes you feel 'out of breath')
**7**	
**8**	**Very Hard** (e.g. the highest level of activity that you can continue doing without stopping)
**9**	
**10**	**Maximal** Effort (e.g. a short burst of activity, such as a sprint, that you cannot maintain for long)

16. Did the participant engage in **aerobic activity of moderate to vigorous intensity** for **at least 150 minutes this week (past 7 days)?**
    - ☐ Yes
    - ☐ No

17. Did the participant engage in **muscle or bone strengthening activities (resistance activities) at least 2 days this week (past 7 days)** (e.g., abdominal lifts, bench press, seated row)?
    - ☐ Yes
    - ☐ No

18. **In the past week, on how many days did the participant do a total of 30 minutes or more of moderate or vigorous physical activity,** which was enough to raise your breathing rate? This may include sport, exercise, and brisk walking or cycling for recreation or to get to and from places, but should not include housework or physical activity that may be part of your job.

    Remember: Moderate-intensity physical activities cause adults to sweat a little and to breathe harder (i.e. brisk walking, bike riding). Moderate-intensity activities can be considered **3, 4 or 5 out of 10** on the RPE scale.
    - ☐ 0 (None)
    - ☐ 1 day
    - ☐ 2 days
    - ☐ 3 days
    - ☐ 4 days
    - ☐ 5 days
    - ☐ 6 days
    - ☐ 7 days

Supplemental File 2: Personal Training Coaching Log for Personal Trainers

19. The following questions refer to **<u>changes in the participant's personal training fitness program</u>** from the last personal training session (2 weeks ago):

	Yes	No
19a) Did the **type of activity (or exercise)** change from the last session?	☐	☐
19b) How did the **type of activity** (or exercise) the participant performed change from the last personal training session (2 weeks ago)?	☐ Added more types of activities this week to the ones already being performed last week.<br>☐ Replaced the activities from last week with new ones this week.<br>☐ Other. Please describe ________	
20a) For each statement, indicate whether the training session **<u>changed</u>** from the last personal training session (2 weeks ago).	**Yes**	**No**
20a)i) Did the **<u>frequency</u>** of exercise (number of repetitions) change from the last session?	☐	☐
20a)ii) Did the **<u>duration</u>** (amount of time) of exercise change from the last session?	☐	☐
20a)iii) Did the **<u>intensity of exercise</u>** change from the last session?	☐	☐

	Increased	Decreased
20b)i) **<u>Frequency</u>** of exercise (number of repetitions)	☐	☐
20b)ii) **<u>Duration</u>** (amount of time) participant exercised	☐	☐
20a)iii) **<u>Intensity</u>** at which participant exercised	☐	☐

Supplemental File 2: Personal Training Coaching Log for Personal Trainers

21. What **types of activity** did the participant engage in **over the past 2 weeks**?

Type of Activity	Yes	No
21a) **Aerobic Activities?**<br>Defined as any activity using large muscle groups, can be maintained continuously and is rhythmic in nature. (e.g., walking, running, bicycling, elliptical etc.)	☐	☐
21b) **Resistance or Strength Training Activities?**<br>Defined as: exercise that involves strengthening of muscle tissue (e.g., bench press, seated row, abdominal lifts, etc.)	☐	☐
21c) **Flexibility Activities?**<br>Defined as stretching or a repeated movement through a joint's complete range of motion. (e.g., stretching of upper and/or lower body)	☐	☐
21d) **Neuromotor or Balance Activities?**<br>Defined as training that incorporates various motor skills. (e.g. balance training, coordination exercises, etc.)	☐	☐

The following questions refer to the participant's use of their Fitbit (or other wireless physical activity monitor) this past week (7 days):

22a) Does the participant have a **Fitbit Inspire** with the CBE Study?

☐ Yes – they have a Fitbit provided with the CBE Study and they used it to track their physical activity this past week

☐ No – but they used their own personal Fitbit (or other wireless physical activity monitor) to track their physical activity this past week. Please indicate type of device: __________

☐ Yes – they have a Fitbit provided with the CBE Study but they did NOT use it to track their physical activity this past week

☐ No – they do not have a Fitbit (or other wireless physical activity monitor). Comments: _______

22b. What were the **total** **number of steps taken** this past week (7 days) as measured by their Fitbit (or other activity monitor)?

Number of steps taken: ___

22c. What was the **total** **distance walked** (also includes jogging or running) this past week (7 days) as measured by their Fitbit Inspire (or other activity monitor)?

Distance in kilometres (km): __

Supplemental File 2: Personal Training Coaching Log for Personal Trainers

22d. Did the participant **sync** their Fitbit this week?

- ☐ Yes
- ☐ No
- ☐ Not applicable – the participant does not have a Fitbit as part of the study

23a) Did the participant encounter any **technology challenges** over the past 2 weeks that impacted their participation in the study (e.g. taking part in the training session online, group YMCA virtual classes, Zoom issues, etc.)?

- ☐ Yes
- ☐ No

23b) Please describe the technology challenges. ___________________________

24. Do you have any other comments to add about the training session or the participant's engagement in the study?

Text Box here….

25. Have you updated the participant's **assigned training program in Virtuagym** with any changes since the previous coaching session? Note: This is an important step to ensure the research team has access to the data they need.

- ☐ Yes
- ☐ No

26. What best describes the **training program** that has been assigned to the participant?

- ☐ Level 1 (Beginner) with adaptations
- ☐ Level 2 (Intermediate)
- ☐ Level 2 (Intermediate) with adaptations
- ☐ Level 3 (Expert)
- ☐ Level 3 (Expert) with adaptations
- ☐ Other – Please describe: ______________________________

[For Baseline Session #1 Only]

13. Do you have any other comments to add about the training session or the participant's engagement in the study?

Text Box here….

14. Have you uploaded / inputted the participant's **assigned training program into Virtuagym**?
Note: This is an important step to ensure the research team has access to the data they need.

- ☐ Yes
- ☐ No
