# Supplementary material for: Fidelity of an Online Telecoaching Community-Based Exercise Intervention Among Adults Living With HIV in Toronto, Ontario, Canada: Observational Longitudinal Study
Source: JMIR Rehabil Assist Technol. 2026 Sep 3;13:e84902. doi: 10.2196/84902 (PMC13586703; doi:10.2196/84902)
Supplement: Supplementary file 3 [file rehab_v13i1e84902_app3.pdf]

Supplemental File 3: Fidelity of Implementation (Criterion 3) - Structured interviews Sensitivity Analyses with adults living with HIV for all participants who initiated (n=32) the study and participants who completed an interview at month 2 (n=27) and month 6 (n-18).

	**Month 2 (n= 27)**			**Entire Sample Month 2 (n=32)**	**Month 6 (n= 18)**			**Entire Sample Month 6 (n=32)**
Fidelity of Implementation Item	N (%; 95% CI) who reported "no criteria met" (0)	N (%; 95% CI) who reported "partial criteria met" (1)	N (%; 95% CI) who reported "complete criteria met" (2)	N (%; 95% CI) who reported "complete criteria met" (2)	N (%; 95% CI) who reported "no criteria met" (0)	N (%; 95% CI) who reported "partial criteria met" (1)	N (%; 95% CI) who reported "complete criteria met" (2)	N (%; 95% CI) who reported "complete criteria met" (2)
	**External Factor***							
1. Participant completed their session in the allotted time (1 hour with coach online)	0 (0%)	6 (22%; 7, 38)	21 (78%; 62, 94)	21 (66%; 49, 82)	0 (0%)	4 (22%; 3, 41)	14 (78%; 59, 97)	14 (44%; 27, 61)
	**Fitness Trainer Factors: The fitness trainer's performance during session***							
2. Fitness trainer provided direct and clear instructions during exercise session	0 (0%)	0 (0%)	27 (100%)	27 (84%; 71, 97)	2 (11%; 0, 26)	0 (0%)	16 (89%; 74, 100)	16 (50%; 33, 67)
3. Fitness trainer interacted with participant in a way that was meaningful or helpful for their session (e.g. provided eye-to-eye contact, feedback, ongoing communication, etc.)	0 (0%)	2 (7%; 0, 17)	25 (93%; 83, 100)	25 (78%; 64, 92)	0 (0%)	2 (11%; 0, 26)	16 (89%; 74, 100)	16 (50%; 33, 67)
4. Fitness trainer instructed participant to complete aerobic exercises according to their set exercise program	2 (7%; 0, 17)	3 (11%; 0, 23)	22 (82%; 67, 96)	22 (69%; 53, 84)	3 (17%; 0, 34)	4 (22%; 3, 41)	11 (61%; 39, 84)	11 (34%; 18, 51)

Supplemental File 3: Fidelity of Implementation (Criterion 3) - Structured interviews Sensitivity Analyses with adults living with HIV for all participants who initiated (n=32) the study and participants who completed an interview at month 2 (n=27) and month 6 (n-18).

	**Month 2 (n= 27)**			**Entire Sample Month 2 (n=32)**	**Month 6 (n= 18)**			**Entire Sample Month 6 (n=32)**
Fidelity of Implementation Item	N (%; 95% CI) who reported "no criteria met" (0)	N (%; 95% CI) who reported "partial criteria met" (1)	N (%; 95% CI) who reported "complete criteria met" (2)	N (%; 95% CI) who reported "complete criteria met" (2)	N (%; 95% CI) who reported "no criteria met" (0)	N (%; 95% CI) who reported "partial criteria met" (1)	N (%; 95% CI) who reported "complete criteria met" (2)	N (%; 95% CI) who reported "complete criteria met" (2)
5. Fitness trainer instructed participant to complete resistive exercises according to their set exercise program	1 (4%; 0, 11)	0 (0%)	26 (96%; 89, 100)	26 (81%; 68, 95)	2 (11%; 0, 26)	3 (17%; 0, 34)	13 (72%; 52, 93)	13 (41%; 24, 58)
6. Fitness trainer instructed participant to complete neuromotor/balance exercises according to their set exercise program	1 (4%; 0, 11)	1 (4%; 0, 11)	25 (93%; 83, 100)	25 (78%; 64, 92)	4 (22%; 3, 41)	2 (11%; 0, 26)	12 (67%; 45, 89)	12 (38%; 21, 54)
7. Fitness trainer instructed participant to complete flexibility exercises according to their set exercise program	0 (0%)	3 (11%; 0, 23)	24 (89%; 77, 100)	24 (75%; 60, 90)	3(17%; 0, 34)	2 (11%; 0, 26)	13 (72%; 52, 93)	13 (41%; 24, 58)
8. Fitness trainer changed the program from past sessions to progress participant activity (either increase or decrease in intensity)	0 (0%)	1 (4%; 0, 11)	26 (96%; 89, 100)	26 (81%; 68, 95)	1 (6%; 0, 16)	0 (0%)	17 (94%; 84, 100)	17 (52%; 26, 70)

Supplemental File 3: Fidelity of Implementation (Criterion 3) - Structured interviews Sensitivity Analyses with adults living with HIV for all participants who initiated (n=32) the study and participants who completed an interview at month 2 (n=27) and month 6 (n-18).

	**Month 2 (n= 27)**			**Entire Sample Month 2 (n=32)**	**Month 6 (n= 18)**			**Entire Sample Month 6 (n=32)**
Fidelity of Implementation Item	N (%; 95% CI) who reported "no criteria met" (0)	N (%; 95% CI) who reported "partial criteria met" (1)	N (%; 95% CI) who reported "complete criteria met" (2)	N (%; 95% CI) who reported "complete criteria met" (2)	N (%; 95% CI) who reported "no criteria met" (0)	N (%; 95% CI) who reported "partial criteria met" (1)	N (%; 95% CI) who reported "complete criteria met" (2)	N (%; 95% CI) who reported "complete criteria met" (2)
	**Participant Factors: Participant engagement and experience***							
9. Participant understood the instructions provided by their coach	0 (0%)	2 (7%; 0, 17)	25 (93%; 83, 100)	25 (78%; 64, 92)	2 (11%; 0, 26)	1 (6%; 0, 16)	15 (83%; 66, 100)	15 (47%; 30, 64)
10. Participant accepted direction/feedback provided by the coach	0 (0%)	0 (0%)	27 (100%)	27 (84%; 71, 97)	2 (11%; 0, 26)	0 (0%)	16 (89%; 74, 100)	16 (50%; 33, 67)
11. Participant engaged in the prescribed aerobic exercises during the most recent session	2 (7%; 0, 17)	3 (11%; 0, 23)	22 (82%; 67, 96)	22 (69%; 53, 84)	3(17%; 0, 34)	6 (33%; 12, 55)	9 (50%; 27, 73)	9(28%; 13, 44)
12. Participant engaged in the prescribed resistive exercises during the most recent session	1 (4%; 0, 11)	1 (4%; 0, 11)	25 (93%; 83, 100)	25 (78%; 64, 92)	2 (11%; 0, 26)	4 (22%; 3, 41)	12 (67%; 45, 89)	12 (38%; 21, 54)
13. Participant engaged in the prescribed neuromotor/balance exercises	1 (4%; 0, 11)	1 (4%; 0, 11)	25 (93%; 83, 100)	25 (78%; 64, 92)	4 (22%; 3, 41)	3 (17%; 0, 34)	11 (61%; 39, 84)	11 (34%; 18, 51)

Supplemental File 3: Fidelity of Implementation (Criterion 3) - Structured interviews Sensitivity Analyses with adults living with HIV for all participants who initiated (n=32) the study and participants who completed an interview at month 2 (n=27) and month 6 (n-18).

	**Month 2 (n= 27)**			**Entire Sample Month 2 (n=32)**	**Month 6 (n= 18)**			**Entire Sample Month 6 (n=32)**
Fidelity of Implementation Item	N (%; 95% CI) who reported "no criteria met" (0)	N (%; 95% CI) who reported "partial criteria met" (1)	N (%; 95% CI) who reported "complete criteria met" (2)	N (%; 95% CI) who reported "complete criteria met" (2)	N (%; 95% CI) who reported "no criteria met" (0)	N (%; 95% CI) who reported "partial criteria met" (1)	N (%; 95% CI) who reported "complete criteria met" (2)	N (%; 95% CI) who reported "complete criteria met" (2)
14. Participant engaged in the prescribed flexibility exercises	1 (4%; 0, 11)	1 (4%; 0, 11)	25 (93%; 83, 100)	25 (78%; 64, 92)	3 (17%; 0, 34)	3 (17%; 0, 34)	12 (67%; 45, 89)	12 (38%; 21, 54)
	**Additional Factors: The broader tele-coaching study intervention**							
15. Participant completed additional individual sessions (60 min 2x/week) this past week	5 (19%; 4, 33)	9 (33%; 15, 51)	13 (48%; 29, 67)	13 (41%; 24, 58)	4 (22%; 3, 41)	3 (17%; 0, 34)	11 (61%; 39, 84)	11 (34%; 18, 51)
16. Participant responded/ completed the most recent weekly exercise email/text message	6 (22%; 7, 38)	1 (4%; 0, 11)	20 (74%; 58, 91)	20 (63%; 45, 79)	4 (22%; 3, 41)	2 (11%; 0, 26)	12 (67%; 45, 89)	12 (38%; 21, 54)
17. Participant attended weekly group-based classes online at the YMCA	15 (56%; 37, 74)	4 (15%; 1, 28)	8 (30%; 13, 47)	8 (25%; 10, 40)	8 (44%; 21, 67)	4 (22%; 3, 41)	6 (33%; 12, 55)	6 (19%; 5, 32)
18. Participant engaged in monthly self-management sessions online	5 (19%; 4, 33)	10 (37%; 19, 55)	12 (45%; 26, 63)	12 (38%; 21, 54)	7 (39%; 16, 61)	7 (39%; 16, 61)	4 (22%; 3, 41)	4 (13%; 1, 24)

Supplemental File 3: Fidelity of Implementation (Criterion 3) - Structured interviews Sensitivity Analyses with adults living with HIV for all participants who initiated (n=32) the study and participants who completed an interview at month 2 (n=27) and month 6 (n-18).

	**Month 2 (n= 27)**			**Entire Sample Month 2 (n=32)**	**Month 6 (n= 18)**			**Entire Sample Month 6 (n=32)**
Fidelity of Implementation Item	N (%; 95% CI) who reported "no criteria met" (0)	N (%; 95% CI) who reported "partial criteria met" (1)	N (%; 95% CI) who reported "complete criteria met" (2)	N (%; 95% CI) who reported "complete criteria met" (2)	N (%; 95% CI) who reported "no criteria met" (0)	N (%; 95% CI) who reported "partial criteria met" (1)	N (%; 95% CI) who reported "complete criteria met" (2)	N (%; 95% CI) who reported "complete criteria met" (2)
19. Participant wore Fitbit regularly	0 (0%)	3 (11%; 0, 23)	24 (89%; 77, 100)	24 (75%; 60, 90)	0 (0%)	0 (0%	18 (100%)	18 (56%; 39, 73)
20. Participant managed the required technology (e.g., Zoom, SweatforGood app, etc)	0 (0%)	12 (44%; 26, 63)	15 (55%; 36, 74)	15 (46%; 30, 64)	1 (5%; 0, 16)	7 (39%; 16, 61)	10 (56%; 33, 79)	10 (31%; 15, 47)

Green shading indicates at least 80% of participants 'completely met' the criteria. 'Fitness Trainer' is analogous to 'Personal Trainer'; * Participants were asked about their experiences at their most recent personal training session.
